# Above the Invasive and Ornamental Attributes of the Traveler’s Palm: An In Vitro and In Silico Insight into the Anti-Oxidant, Anti-Enzymatic, Cytotoxic and Phytochemical Characterization of *Ravenala madagascariensis*

**DOI:** 10.3390/antiox12010184

**Published:** 2023-01-12

**Authors:** Shanoo Suroowan, Eulogio Jose Llorent-Martínez, Gokhan Zengin, Stefano Dall’Acqua, Stefania Sut, Kalaivani Buskaran, Sharida Fakurazi, Bao Le Van, Mohnad Abdalla, Ashraf N. Abdalla, Asaad Khalid, Mohamad Fawzi Mahomoodally

**Affiliations:** 1 Department of Health Sciences, Faculty of Medicine and Health Sciences, University of Mauritius, Réduit 80837, Mauritius; 2 Department of Physical and Analytical Chemistry, University of Jaén, Campus Las Lagunillas S/N, E-23071 Jaén, Spain; 3 Department of Biology, Science Faculty, Selcuk University, Konya 42130, Turkey; 4 Department of Pharmaceutical and Pharmacological Sciences, University of Padova, Via Marzolo 5, 35131 Padova, Italy; 5 Laboratory of Natural Medicine and Product Research, Institute of Bioscience, Universiti Putra Malaysia, Serdang 43400, Selangor Darul Ehsan, Malaysia; 6 Department of Human Anatomy, Faculty of Medicine and Health Sciences, Universiti Putra Malaysia, Serdang 43400, Selangor Darul Ehsan, Malaysia; 7 Institute of Research and Development, Duy Tan University, Da Nang 550000, Vietnam; 8 Faculty of Natural Sciences, Duy Tan University, Da Nang 550000, Vietnam; 9 Pediatric Research Institute, Children’s Hospital Affiliated to Shandong University, Jinan 250022, China; 10 Department of Pharmacology and Toxicology, College of Pharmacy, Umm Al-Qura University, Makkah 21955, Saudi Arabia; 11 Substance Abuse and Toxicology Research Center, Jazan University, P.O. Box 114, Jazan 45142, Saudi Arabia; 12 Medicinal and Aromatic Plants and Traditional Medicine Research Institute, National Center for Research, Khartoum P.O. Box 2404, Sudan; 13 Center for Transdisciplinary Research, Department of Pharmacology, Saveetha Dental College, Saveetha Institute of Medical and Technical Science, Chennai 600077, India; 14 Centre of Excellence for Pharmaceutical Sciences, North-West University, Private Bag X6001, Potchefstroom 2520, South Africa

**Keywords:** *Ravenala madagascariensis*, antioxidants, enzyme inhibition, natural agents

## Abstract

*Ravenala madagascariensis* is a widely known ornamental and medicinal plant, but with a dearth of scientific investigations regarding its phytochemical and pharmacological properties. Hence, these properties were appraised in this study. The DPPH (154.08 ± 2.43 mgTE/g), FRAP (249.40 ± 3.01 mgTE/g), CUPRAC (384.57 ± 1.99 mgTE/g), metal chelating (29.68 ± 0.74 mgEDTAE/g) and phosphomolybdenum assay (2.38 ± 0.07 mmolTE/g) results demonstrated that the aqueous extract had the most prominent antioxidant activity, while the methanolic extract displayed the best antioxidant potential in the ABTS assay (438.46 ± 1.69 mgTE/g). The HPLC-ESI-Q-TOF-MS-MS analysis allowed the characterization of 41 metabolites. The methanolic extract was the most active against acetylcholinesterase. All extracts were active against the alpha-amylase and alpha-glucosidase enzymes, with the ethyl acetate extract being the most active against the alpha-amylase enzyme, while the methanolic extract showed the best alpha-glucosidase inhibition. A plethora of metabolites bonded more energetically with the assayed enzymes active sites based on the results of the in silico studies. *R. madagascariensis* extracts used in this study exhibited cytotoxicity against HT29 cells. The IC_50_ of the methanolic extract was lower (506.99 ug/mL). Based on the heat map, whereby flavonoids were found to be in greater proportion in the extracts, it can be concluded that the flavonoid portion of the extracts contributed to the most activity.

## 1. Introduction

*Ravenala madagascariensis*, also known as the traveler’s palm or the traveler’s tree, is endemic to Madagascar and grows well in Africa, the Americas, Asia and Australia [1,2]. This tree is widely used as an ornamental plant throughout the tropics and finds multipurpose utilities in Madagascar and other African countries, such as for its uses in construction, fencing, and food [3,4]. The plant is cultivated, and also harvested from the wild. The plant parts are utilized for roofs, walls, and floors in homes. In India, the plant is used to build houses, and leaves are used as packing material for roofing. Other parts are used to build hut walls [2].

Alongside these, it is traditionally employed to treat various diseases, such as diabetes and tooth decay [4,5]. In Mauritius, *R. madagascariensis* is an invasive plant species and is a threat to the native forests.

In studies on the various applications of the *R. madagascariensis*, it was revealed that its aerial parts have antimicrobial properties, and its leaf extract has antioxidant properties [6,7]. Ethnobotanical studies demonstrate its uses for a panoply of ailment conditions, such as against coughs, stomachache, urine retention, diabetes, diarrhea, edema, kidney stones and hypertension [8,9].

Despite *R. madagascariensis* being widely employed traditionally as traditional medicine, there are not many studies that have investigated in detail its phytochemical composition and biological activities through rigorous methods of investigation. Previous in vitro investigations have mostly been oriented towards studying in vitro the properties of the plant as antidiabetic, antithrombolytic and as an antimicrobial [6,7,10,11].

The rich ethnomedicinal background of *R. madagascariensis*, coupled with its invasive properties and poor scientific evaluation, renders this plant species a potential candidate for the evaluation of its pharmacological properties in an attempt to discover cost-effective novel leads/extracts or medicines with noticeable therapeutic properties.

One of the most challenging tasks when studying plant species remains the isolation and identification of plant phytochemicals. Over the years, diverse methods of phytochemical screening techniques have been developed and used successfully for this purpose, but one of the most established and continuously innovating methods remains liquid chromatography-mass spectrometry. Chromatographic methods of plant screening remain the most used methods of determination of plant composition [12].

To complement the phytochemical fingerprinting of *R. madagascariensis*, various assays were conducted in this study. The antioxidant, antidiabetic, anticancer, antityrosinase, and metal chelating properties, and its potential against neurodegenerative disorders such as Alzheimer’s disease, were also investigated in vitro.

In an attempt to enhance the interpretation of the enzyme inhibitory activities, in silico docking studies of the phytoconstituents whose structure is available on PubChem was also performed. Indeed, computational docking is a routinely employed method for the determination of protein-ligand interactions to discover and develop new drugs [13].

## 2. Materials and Methods

The leaves of *Ravenala madagascariensis* Sonn., were collected from MonVert nature park located in the district of Plaine Wilhems, Curepipe, Mauritius island, under the supervision of the resident botanist. Samples (fruits, flowers and leaves) of the collected plant species were deposited at the Mauritius Herbarium at the Mauritius Sugarcane Industry and Research Institute (MSIRI) situated in Réduit, Mauritius for validation of their identity. The identity of *Ravenala madagascariensis* Sonn. was confirmed from the deposited samples. The identified plant species were assigned the following barcode numbers: *Ravenala madagascariensis* Sonn.: MAU0027517.

### 2.1. Extraction of Phytochemicals

The leaves of the plant were carefully cleaned using a combination of water and distilled water to remove debris. They were then kept in a well-ventilated area and protected from the sun. A daily analysis of the leaves revealed that their mass had gradually decreased over the course of three weeks. The dried leaves were then subjected to a mechanical grinder. After they had been thoroughly cleaned, the leaves were placed in a beaker and subjected to macerating at room temperature and pressure. The dried plant components were then individually placed in a liter of methanol, ethyl acetate and distilled water. This process lasted for about 14 days. After the beakers had been shaken constantly, the leaves were then filtered using Whatman^®^ filter paper.

A decoction was then prepared by adding 50 g of dried *R. madagascariensis* powder to 200 mL of water. At a temperature of about 100 °C, the mixture was boiled until it was reduced to a quarter of its original volume. The resulting mixture was then filtered through a cloth. The organic and aqueous filtrates were then combined in a rotary evaporator at a pressure and temperature of 4 °C. The crude extract was then subjected to in vitro and phytochemical screening.

### 2.2. Phytochemical Composition

The total phenolic, flavonoid, and acid content, as well as the total phenolic acid, were determined using colorimetric methods. These compounds were expressed as mg of rutin, caffeic acid, gallic acid, and catechin per g of dried extract. These were determined using methods employed in previous studies [14,15].

### 2.3. HPLC-ESI-Q-TOF-MS-MS Analysis

Detailed chromatographic conditions are available in [16] and are also given in the Supplementary Information.

### 2.4. Biological Activities Evaluation

#### 2.4.1. Antioxidant Assays

A wide range of antioxidant tests were performed, such as radical scavenging, metal chelation, and phosphomolybdenum reduction. Enzyme inhibitory activities, such as the ChE, Elmann’s method, alpha-amylase, dopachrome, and alpha-glucosidase, were also determined using the methods described in Zengin et al. [15]. The radical scavenging activity of DPPH was analyzed using the method described in this article. A sample solution containing 1 mg/mL of DPPH was added to a methanol solution containing 0.004% DPPH. The absorbance at 517 nm was obtained after 30 min of incubation at room temperature. The radical scavenging activity was then measured as mg TE/g extract.

The ABTS+ radical scavenging assay was performed by reacting 7 to 8 mM ABTS solution with a potassium persulfate solution of 2.45 to 3.4 mM. For 12–16 min at room temperature, the mixture was kept in a dark state. Before the start of the test, the solution was diluted with methanol. This resulted in an absorbance of 0.700 to 0.02 at 734 nm. A sample solution containing 1 mg/mL of DPPH was then added to the ABTS solution and mixed. The absorbance at 734 nm was obtained after 30 min of incubation. The radical scavenging activity of the ABTS solution was then measured as mgTE/g extract [17]. The CUPRAC activity was measured using a sample solution that was added to a premixed reaction mixture that included NH_4_Ac buffer, neocuproine, and CuCl_2_. A blank was then prepared by adding a sample solution that was 0.5 mL to the mixture without CuCl_2_.

The absorbances of the sample and the blank were measured at 450 nm after 30 min of incubation at room temperature. The CUPRAC activity was then calculated as mg TE/g extract.

The FRAP activity was then measured using a sample solution that was added to a premixed reaction mixture that included 2,4,6-tris (2-pyridyl)-S-triazine and 0.1 mL of acetate buffer. The mixture was then subjected to 40 mM HCl and ferric chloride in a ratio of 10:1. After 30 min of incubation, the absorbance of the sample was measured at 593 nm. The activity was then expressed as mg TE/g extract. The purpose of the phosphomolybdenum analysis was to determine the total antioxidant capacity of a sample solution prepared by adding a premixed reaction mixture consisting of 0.6 M sulfuric acid, 28 M sodium phosphate, and 4 M ammonium molybdate. The absorbance of the sample at 695 nm was obtained after 90 min of incubation [17].

The metal cation channel activity was then measured using a sample solution that was added to a premixed reaction mixture that included 5 mM ferrozine. A blank was then prepared by adding a sample solution containing 2 mL of FeCl_2_ and water. The absorbances of the sample and the blank were measured at 562 nm after 10 min of incubation at room temperature. The metal cation channel activity was then measured using a sample solution that was added to a premixed reaction mixture that included 5 mM EDTA. The activity was then expressed as milligrams of the extracted mg TE/g extract.

#### 2.4.2. Anticholinesterase Activities

The activity of the cation channel was then measured using a sample solution that was added to a premixed reaction mixture that included 5,5-dithio-bis(2-nitrobenzoic acid) and 5,5′-dithio-bis-(2-nitrobenzoic acid) (DTNB). It was then subjected to a 96-well microplate containing a Tris-HCl buffer at a temperature of 25 degrees Celsius for 15 min. The reaction was initiated with the addition of butyrylthiocholine chloride or acetylthiocholine iodide (25 µL). A blank was then prepared by adding a sample solution to the reaction reagents that did not contain an enzyme. The absorbances of the sample and the blank were measured at 405 nm after 10 min of incubation at 25 degrees Celsius. The absorbance of the blank was then taken into account with respect to the sample. The inhibitory activity of the cholinesterase was then calculated as mgGALAE/g [17].

#### 2.4.3. Anti-Tyrosinase Activities

The activity of the cation channel was then measured using a sample solution that was added to a premixed reaction mixture that included 5,5-dithio-bis(2-nitrobenzoic acid) and 5,5′-dithio-bis-(2-nitrobenzoic acid) (DTNB). It was then subjected to a 96-well microplate at a temperature of 25 degrees Celsius for 15 min. A blank was then prepared by adding a solution containing an enzyme-free reaction mixture to all the reaction reagents. The absorbances of the sample and the blank were measured at 492 nm after 10 min of incubation at 25 degrees Celsius. The absorbance of the blank was then taken into account and the inhibitory activity of the tyrosinase was calculated as the kojic acid equivalents of the extracted mg KAE/g extract [17].

#### 2.4.4. Alpha-Amylase and Alpha-Glucosidase Assays

The activity of the alpha-amylase was measured using a sample solution that was added to a premixed reaction mixture that included 5,5-dithio-bis(2-nitrobenzoic acid) and DTNB. It was then subjected to a 96-well microplate at a temperature of 25 degrees Celsius for 10 min. The reaction was then initiated after pre-incubation. A blank was then prepared by adding a solution containing an enzyme-free reaction mixture to all the reaction reagents. The mixture was then incubated at 37 degrees Celsius for 10 min. The reaction was stopped after the addition of HCl and iodine-potassium iodide. The absorbances of the sample and the blank were measured at 630 nm. The absorbance of the sample was then taken into account, and the alpha-amylase inhibitory activity was calculated as the acarbose equivalents of the extracted mmol ACAE/g [17].

A sample solution containing 1 mg/mL of alpha-glucosidase was prepared by adding a mixture of alpha-glucosidase solution from *Saccharomyces cerevisiae* and glutathione. It was then subjected to a 96-well microplate at a temperature of 37 degrees Celsius for 15 min. A blank was then prepared by adding a solution containing an enzyme-free reaction mixture to all the reaction reagents. The reaction was stopped after the addition of sodium carbonate. The absorbances of the blank and the sample were measured at 400 nm. The absorbance of the sample was then taken into account and the alpha-amylase inhibitory activity was calculated as the acarbose equivalents of the extracted mgKAE/g extract [17].

### 2.5. Cell Viability Assay

The toxicity level of the extract was analyzed by performing a cell viability test. Three types of cell lines, the (i) normal human fibroblasts (3T3) cells, (ii) human hepatocellular carcinoma cells HepG29, and (iii) human colorectal carcinoma cells, HT29, were used for the test. The cells were purchased from ATCC (Manassas, VA, USA). The cells were grown using Roswell Park Memorial Institute (RPMI) of 1640 medium (Nacalai Tesque, Kyoto, Japan) in which 10% fetal bovine albumin (Sigma-Aldrich, MO, USA) was also added alongside 1% antibiotics containing 10,000 units/mL penicillin and 10,000 μg/mL streptomycin (Nacalai Tesque, Kyoto, Japan). Growth of cells was maintained at the following conditions: humidity (5%), carbon dioxide (95%) and temperature (37 °C). Matured cell layers were collected, employing 0.25% trypsin/1mM-EDTA (Nacalai Tesque, Kyoto, Japan). The procedure ensued by seeding in a 96-well tissue culture plates at 1.0 × 10^4^ cells/well for 24 h in an incubator to attach and 80% confluence was attained for treatment. The methylthiazol tetrazolium (MTT)-based assay was conducted to evaluate the cell viability and cytotoxicity. A stock solution was prepared by mixing in 1:1 of dimethyl sulfoxide (0.1%) and RPMI Cells were treated with this. Various final concentrations were then prepared by further diluting in the same media to produce amounts ranging from 1.25 to 100 μg/mL. Once the cells were attached to the respective wells after 24 h, the tested extracts were added until the final volume of 100 μL per well was obtained. A total of 10 μL of MTT solution (5 mg/mL in PBS) was added to each well and further incubated for 3 h before being aspirated following a period of 72 h of incubation. Then, 100 μL of dimethyl sulfoxide was added per well in the dark and at room temperature in order to dissolve the purple formazan salt. The microplate reader was calibrated at a wavelength of 570 nm to measure the intensity of the purple formazan solution (Biotek LE800, Winooski, VT, USA).

The various cell cytotoxicity tests were performed in triplicate. The standard deviations were then incorporated into the bar graphs. For the calculation of the calculation of IC_50_, the values of the *y*-axis and the *x*-axis were converted to their log values, followed by nonlinear regression (curve fit) under the xy analysis to obtain a straight line equation fit, y = ax + b, from which the regression line and then inhibition IC_50_ were calculated.

### 2.6. In Silico Docking Studies

Crystal structures of studied enzymes were extracted from the protein data bank (PDB) (https://www.rcsb.org/, accessed on 15 December 2022): Cholinesterase enzymes (Acetylcholinesterase: pdb: (PDB:2YDM)) and Butyrylcholinesterase: (PDB: 5HF5); enzymes involved in diabetes pathogenesis (alpha-amylase: (PDB: 1VAH)) and alpha-glucosidase: (PDB: 3AXI); and Tyrosinase: (pdb:2Y9X). The details of the model construction have been described previously [18], as well as the prepared protein structures [18]. The 3D structures of selected ligands were downloaded from the PubChem database (https://pubchem.ncbi.nlm.nih.gov/, accessed on 15 December 2022). The IUPAC name, PubChem database link and PubChem CID are summarized in Supplementary Table S1. The respective cocrystal ligand of each complex was used to define the docking grid box dimension and binding coordinates using AutoDockTools 1.5.6, and docking was performed using AutoDock 4.2.6 (https://autodock.scripts.edu, accessed on 15 December 2022). The docking score of each ligand was calculated, and the protein–ligand interactions were visualized using Biovia Discovery Studio Visualizer (Dassault Systèmes Biovia Software Inc., San Diego, CA, USA, 2012).

### 2.7. Statistical Analysis

Data are presented as mean ± standard deviation of the number (*n* = 3) of replicates. One-way analysis of variance with Tukey’s post hoc test was conducted; *p* < 0.05 was considered statistically significant. The statistical evaluation was performed using Graphpad version 9.0.

## 3. Results

### 3.1. Total Bioactive Components

All extracts of *R. madagascariensis* contained phenolics, flavonoids, phenolic acid and flavonols. The highest phenolic content was noted for the aqueous extract, while the highest flavonoid, total phenolic acid and flavonol content was noted for the methanolic extract. The detailed results of the phytochemical evaluation results are presented in Table 1. The highest phenolic content was noted in the aqueous extract, despite the methanolic extract being highest in flavonoid, phenolic acid and flavonol content. This may be due to the presence of other polyphenolic compounds such as tannins and stilbenes in the aqueous extract.

### 3.2. Characterization of Phytochemicals by HPLC-ESI-Q-TOF-MS-MS

The accurate mass data, MS/MS fragmentation patterns, as well as METLIN database and bibliography information were used for the characterization. Table 2 contains the information of the characterized compounds: retention time, experimental mass, molecular formula, calculated mass error (ppm) and fragment ions. A brief explanation of the identification follows when analytical standards or the METLIN database were not used.

Compound 5 suffered the neutral loss of 162 Da (hexoside moiety) to yield dihydrobenzoic acid at *m*/*z* 109 (confirmed with METLIN database), so it was characterized as dihydroxybenzoic acid-*O*-hexoside.

A high percentage of the identified compounds corresponded to flavonoid glycosides. The aglycones isorhamnetin, kaempferol and quercetin were identified by comparison with analytical standards and the database METLIN. The attached moieties were characterized based on the neutral losses of 162 Da (hexoside), 146 Da (deoxyhexoside), 132 Da (pentoside) and 308 Da (rutinoside). However, the losses of 162 Da and 176 Da in compounds **35**, **37** and **39** were attributed to caffeoyl and feruloyl moieties based on the exact molecular mass. 

Compound **12** was characterized as apigenin-6-*C*-pentoside-8-*C*-hexoside based on its fragmentation pattern [19]

Several proanthocyanidins were also identified. Compounds **15**, **16** and **36** were characterized as dimers of (epi)afzelechin [20,21], whereas compounds **24** and **30** were tentatively characterized as a proanthocyanidin trimer of (epi)afzelechin and (epi)fisetinidol units [21,22].

Compounds 40 and 41 were characterized as the oxylipins oxo-dihydroxy-octadecenoic acid and trihydroxy-octadecenoic acid based on bibliographic information [22].

**Figure 1:**
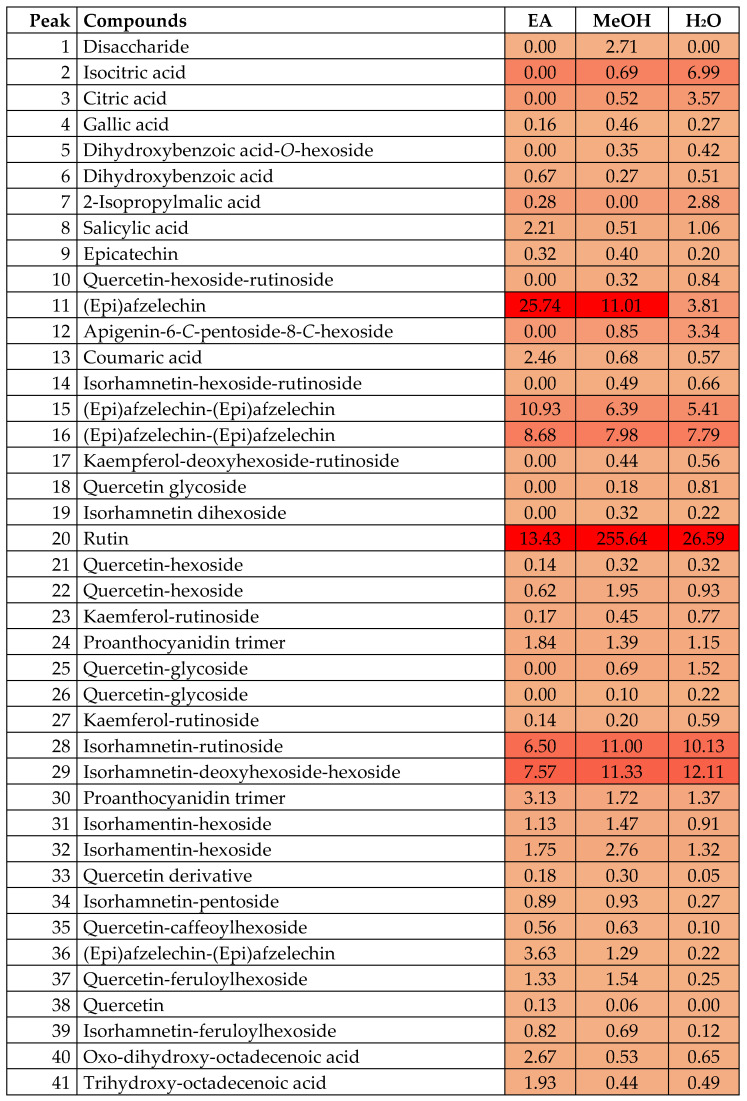
Relative peak areas and heat map obtained by HPLC-Q-TOF-MS-MS analysis of extracts of *R. madagascariensis*. Hex and dHex stand for hexoside and deoxyhexoside, respectively.

As can be seen in Table 2, aqueous and methanol extracts presented a similar profile, whereas the ethyl acetate extract was not as efficient during the extraction of flavonoids. Hence, the higher bioactivity observed in aqueous and methanol extracts is due to the higher abundance of bioactive compounds. We prepared a heat map to check the most abundant compounds in the analyzed extracts (Figure 1). With this purpose, we calculated peak areas for each compound (using MS mode chromatograms, with the corresponding [M-H]- ion for each compound). Then, the relative percentage of each compound was calculated (with regard to the total area of all compounds). In the heat map (Figure 1), the percentage of each compound is given. In addition, to ease its interpretation, the color indicates the relative abundance of each compound (the darker the color, the higher the concentration). The most abundant compounds in methanol and aqueous extracts were flavonoids (approximately 60% of the extract), followed by proanthocyanidins. The main compound in methanol and aqueous extracts was rutin (compound **20**), followed by compounds **28** and **29** (isorhamnetin glycosides) and (epi)afzelechin monomer and dimers (compounds **11**, **15** and **16**). However, the most abundant compounds in each extract can be observed in Figure 1. To prepare this figure, peak areas of each compound were obtained in MS mode using the precursor ion [M-H]-. The relative percentage of each compound was calculated by area normalization. The chemical structures of the main compounds are shown in the Supplementary Information (Figure S1).

The heat map enables the visualization of the most abundant compounds (the darker the color, the higher the concentration). It can be observed that the most abundant compounds in methanol and aqueous extracts were flavonoids (approximately 60% of the extract), followed by proanthocyanidins. Rutin (compound **20**) was the most abundant in methanol and aqueous extracts, followed by compounds **28** and **29** (isorhamnetin glycosides) and (epi)afzelechin monomer and dimers (compounds **11**, **15** and **16**). On the other hand, the ethyl acetate extract presented a different pattern: compounds **11**, **15** and **16** represented 45% of the identified compounds, followed by compounds **20**, **28** and **29**, with the opposite tendency observed in methanol and aqueous extracts. To sum up, it is clear that the bioactivity of the different extracts was determined by the presence of the main flavonoids (rutin and isorhamnetin glycosides) and (epi)afzelechin and its dimers.

The heat map visualizes the data regarding the proportion of metabolites in the different extracts. The more intense color of the numbers of metabolites demonstrates that these metabolites are present in the largest proportion compared to the pale colors. According to the heat map, the disaccharide was present only in the methanolic extract. The aqueous extract had the highest proportion of isocitric, 2-isopropylmalic and citric acid as well as apigenin-6-C-pentoside-8-C-hexoside. (epi)afzelechin, (epi)afzelechin–(epi)afzelechin, rutin, isorhamnetin–rutinoside and isorhamnetin–deoxyhexoside–hexoside were amongst the most abundant phytochemicals in all the three extracts investigated and were found in varying proportions. Detailed quantification of the different metabolites from the three extracts is described in Figure 1.

### 3.3. Antioxidant Properties

Based on the DPPH, FRAP, CUPRAC, metal chelating and phosphomolybdenum assays, the aqueous extract of *R. madagascariensis* had the best antioxidant properties. In six antioxidant assays performed, the aqueous extract exhibited the best antioxidant potential in five assays. The methanolic extract exhibited the most powerful antioxidant activity in the ABTS assay. In each assay where the aqueous extract displayed the best antioxidant potential, it was followed by the methanolic extract and the opposite was noticed for the methanolic extract in the ABTS assay. The ethyl acetate extract was the least active in terms of antioxidant potential, but not far behind the aqueous and methanolic extract. The details results of the antioxidant evaluation of the *R. madagascariensis* extracts are shown in detail in Table 3.

### 3.4. Enzyme Inhibitory Properties

Table 4. The opposite was noticed in the butyrylcholinesterase investigation. The aqueous extract was not active against any of the above-mentioned enzymes. The results of the anti-tyrosinase activities of the extracts demonstrated that the methanolic and ethyl acetate extracts had almost same efficiencies in inhibiting the tyrosinase enzyme, with the ethyl acetate extract being the most active. All extracts were active against the alpha-amylase and alpha-glucosidase enzymes, with the ethyl acetate extract being the most active against the alpha-amylase enzyme, while the methanolic extract showed the best alpha-glucosidase inhibition.

#### Cytotoxic Effects

**Figure 2:**
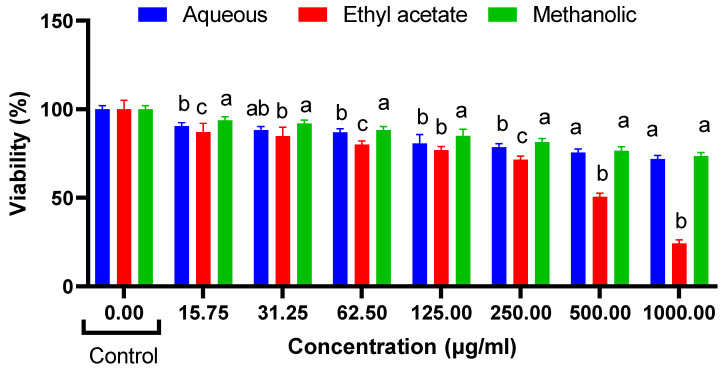
Cell cytotoxicity on NIH 3T3 cells (one-way ANOVA, different letters indicate significant difference between extracts in the same concentration (a, b and c), *p* ≤ 0.05.).

**Figure 3:**
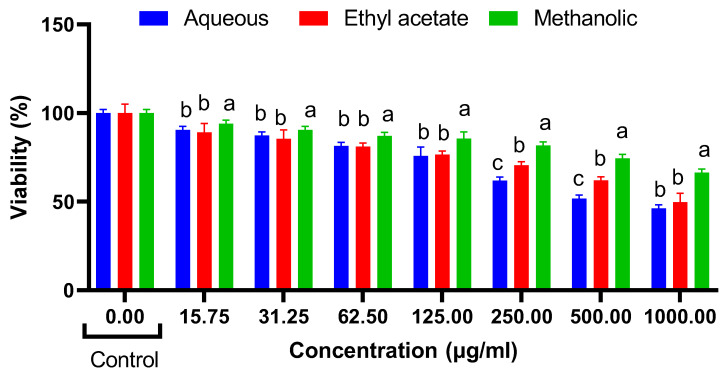
Cell cytotoxicity on HepG2 cells (one-way ANOVA, different letters indicate significant difference between extracts in the same concentration (a, b and c), *p* ≤ 0.05.).

**Figure 4:**
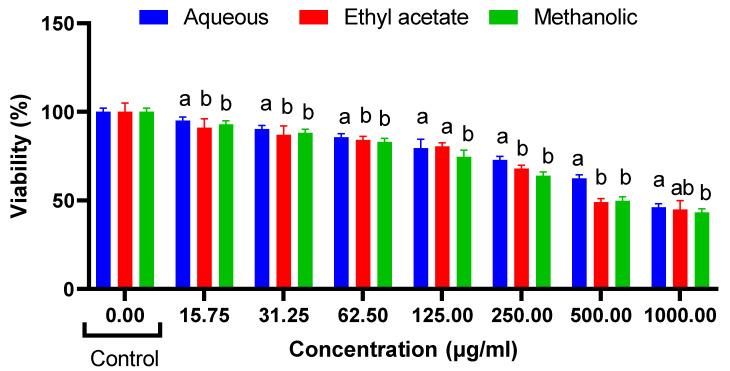
Cell cytotoxicity on HT-29 cells (one-way ANOVA, different letters indicate significant difference between extracts in the same concentration (a, b and c), *p* ≤ 0.05.).

The results of the cytotoxicity assays demonstrate the effects of *R. madagascariensis* aqueous, ethyl acetate and methanolic extract on NIH 3T3, HepG2 and HT29 cells (Figure 2, Figure 3 and Figure 4). Only the ethyl acetate extract demonstrated cytotoxicity against NIH 3T3 cells; the extract was nontoxic up to 250 µg/mL. Upon calculation of the IC_50_ value, it was found to be 503.5 µg/mL. Both the aqueous and the methanolic extracts were non cytotoxic. The detailed illustrations of the effect of *R. madagascariensis* extracts on NIH 3T3 cells is shown in Figure 2. With regard to the cytotoxicity assays conducted on HepG2 cells, the aqueous and ethyl acetate extracts were non-toxic up to 500 µg/mL. The methanolic extract was non cytotoxic. The IC_50_ values of the aqueous and methanolic extracts were 530.88 µg/mL and 988.53 µg/mL, respectively. The detailed results of the effect of *R. madagascariensis* extracts on HepG2 cells are shown in Figure 2. All of the *R. madagascariensis* extracts used in this study exhibited cytotoxicity against HT29 cells. The IC_50_ values were in the order of *R. madagascariensis* methanolic extract 506.99 ug/mL, followed by the ethyl acetate extract (IC_50_ = 538.27) and finally the aqueous extract, whose IC_50_ value was 824.14 ug/mL, respectively. Figure 3 shows in detail the effects of the different extracts on HT29 cells.

### 3.5. In Silico Studies

Docking studies of the identified compounds in *R. madagascariensis* were performed against five enzymes (acetylcholinesterase, butyrylcholinesterase, alpha-amylase, alpha-glucosidase and tyrosinase). All the investigated metabolites could bind to the different enzymes’ active sites with varying binding energies. Interestingly, a number of compounds interacted and bound with the active site with binding energies higher than the control used. Epicatechin, (Epi)afzelechin, quercetin, rutin and isorhamnetin rutinoside bound to the acetylcholine active site with higher energy than the control. All 10 metabolites investigated bound to the active site of alpha-amylase and butyrylcholinesterase enzymes versus the controls used. Only four and five metabolites were more strongly bonded to the active site of alpha-glucosidase and tyrosinase, respectively, compared to the control. The results are shown in Table 5.

With regard to rutin-alpha amylase docking, seven different ligands were involved in binding to the active site following their interaction with different residues through the formation of hydrogen bonds. The most tightly bound ligand was the O8 68 ligand, which was bound to the OE2 receptor by forming a hydrogen bond with the GLU 33 (A) residue and the binding energy recorded was −4.4 kcal/mol.

**Figure 5:**
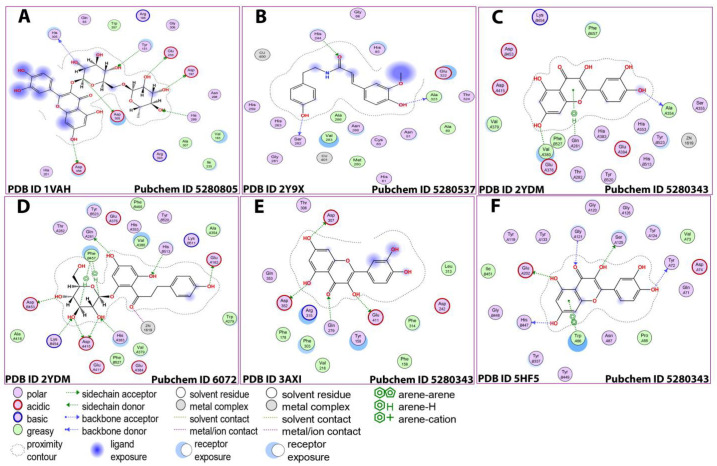
Results of detailed docking (**A**–**F**).

**Figure 6:**
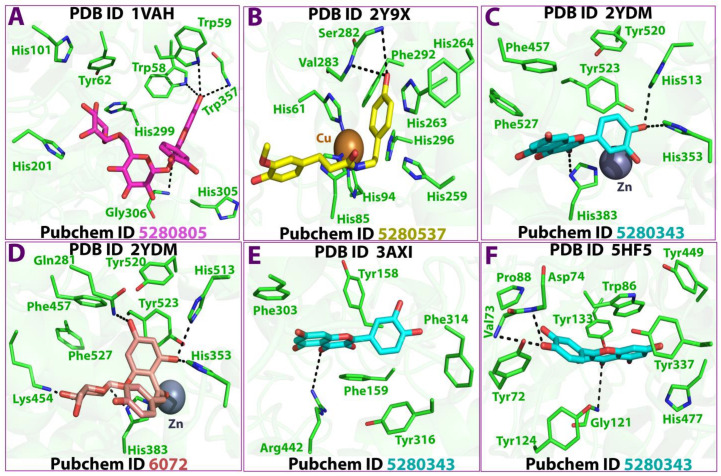
Bond formation during docking (**A**–**F**).

The binding of acetylcholine and quercetin can be possible through the binding of four different ligands with different receptors of the enzyme. The strongest interaction (binding energy = −21.6 kcal/mol) occurred between the Zn 1619 ligand Glu 384 residues of the OE2 receptor through the formation of an ionic bond. Similarly, the strongest binding between quercetin and alpha-glucosidase and butyrylcholinesterase occurred among the 05 31 ligand and OD 2 receptor by binding to ASP 307 (A) residues of the enzyme through the formation of a hydrogen bond (binding energy = −4.9 kcal/mol). The strongest interactions (binding energy = −2.6 kcal/mol) occurred between the O2 27 and O5 31 ligands and Ser 125 (A) and His 447 (A) residues of the OG and O receptors, respectively, through the formation of hydrogen bonds (Table 6 and Figure 5 and Figure 6).

## 4. Discussion

*Ravenala madagascariensis* Sonn. remains an invasive species found in northern America, the tropics and the subtropics [23]. Given its invasiveness, easy availability and long-standing use in traditional systems of medicine, its phytochemical composition and biological properties deserve scrutiny for the purpose of finding affordable plant-based extracts with therapeutic values at the reach of everyone. Its cultivation in various places as an ornamental species renders access to this plant species even more easy.

Despite this species being generally well-known, only a few studies have ventured to study its medicinal virtues. In vitro and in vivo investigations on this plant species have been geared towards studying its antidiabetic, antimicrobial and anti-thrombolytic properties [6,7,10]. We, therefore, aimed to study the antioxidant, enzyme inhibitory potential (anti-alpha amylase, anti-alpha-glucosidase, anti-cholinesterase, anti-butyrylcholinesterase, anti-tyrosinase) activities of *R. madagascariensis*. The cytotoxicity potential against three cell lines (NIH 3T3, HepG2 and HT29 cells) was also investigated.

For the antioxidant potential determination, more than one assay was employed. Indeed, the antioxidant potential of the leaf extracts of *R. madagascariensis* was determined through six different assays. The conductance of different antioxidant assays allows various levels of validation of the antioxidant potential of the extracts.

In five different assays (DPPH, FRAP, CUPRAC, metal chelating and phosphomolybdenum assays), the aqueous extract exerted the best antioxidant potential. Only in the ABTS assay was the methanolic extract antioxidant effect slightly higher than for the aqueous extract. In the other assays, despite the aqueous extract exhibiting the best antioxidant activities, the results for the methanolic extract were very close to that of the aqueous extract, demonstrating that both extracts have noticeable antioxidant properties. The ethyl acetate extract also exhibited antioxidant effects to a certain extent as per the results obtained in the different assays performed.

As per the HPLC analysis, out of the 41 compounds characterized from the *R. madagascariensis* extracts, 38 were common in both aqueous and methanolic extracts. This could explain the fact that both extracts exhibited close antioxidant effects, while the ethyl acetate extract contained only 73% of the compounds characterized and may, hence, account for the lowered antioxidant effect observed.

Peak areas as per the heat map of isocitric acid, citric acid, 2-Isopropylmalic acid, rutin, and Isorhamnetin-deoxyhexoside-hexoside, among others, were higher in the aqueous extract vs. the methanolic extract. Isocitric acid has been identified as a powerful antioxidant, and modern investigations focus on how to increase its yield since it is difficult to synthesize [24]. Citric acid has also been earmarked as possessing noticeable antioxidant properties [25,26]. 2-Isopropylmalic acid has been found to be present in red wine and to exert mild antioxidant activities, having an EC50 of >4800 mg/L in DPPH assays [27].

Rutin is commonly referred to as vitamin P or rutoside, and it is known to have a plethora of pharmacological properties. It has cardio and neuro protective activities and has been found to exert cytotoxicity in a plethora of cancer cell lines. It also exerts antioxidant, anticarcinogenic, cytoprotective, and neuroprotective properties, among other effects. It is widely found in apples and contributes majorly to the beneficial effects of this fruit, whereby it greatly contributes to validate the saying “an apple a day keeps the doctor away” [28].

In furtherance, the HPLC analysis demonstrated the presence of various secondary metabolites that are known to possess diverse pharmacological properties. Gallic acid identified in the extracts is known to exert anti-inflammatory properties by acting through the MAPK and NF-KB signaling pathways. Through its action on these pathways, the release of inflammatory mediators such as adhesion molecules, cytokines and chemokines, among others, is by default dwindled [29].

Salicylic acid (SA) suppresses the transcription of genes which result into the bio-synthesis of cyclooxygenases. SA is also known to inhibit oxidative stress and prostaglandin synthesis, and it is confirmed that it can bind iron as well [30].

Epicatechin is present in various foods we consume every day. It is associated with noteworthy anti-inflammatory and anti-oxidant properties, and also contributes to cardiovascular and cerebral health as well as improving muscle performance [31].

The polyphenolic compound quercetin is a multifaceted pharmacological agent. It inhibits oxidative stress both in vivo and in vitro, is a potent anticancer candidate causing the inhibition of cancer cell growth, and can retard the onset of Alzheimer’s disease through its inhibitory action on acetylcholinesterase enzyme [32].

Kaempferol, alongside its glycosylated derivatives, is known to have antidiabetic, anti-inflammatory, antioxidant, antimicrobial, antitumor and anticancer, cardioprotective and neuroprotective activities, among others [33].

The combined presence of these phytochemicals in higher amounts in the aqueous extract versus the methanolic extract, coupled with the synergistic properties of other metabolites, may account for the heightened antioxidant potential of the aqueous extract. However, given that the same metabolites are also present in the methanolic extract, but in slightly lower proportions, this may account for the close antioxidant efficacies of both extracts.

Seventy five percent of cases of dementia are due to Alzheimer’s disease (AD). Cholinergic impairment is responsible for the progression of the disease and, hence, cholinesterase enzyme inhibitors have become the ideal candidates in AD therapy. Among the inhibitors of acetylcholine, galanthamine is the sole naturally occurring one. Hence, there is plenty of room left for the discovery of other sources of cholinesterase enzyme inhibitors for the proper management of the disease and the retardment of disease progression [34].

Diverse plant families are known to be good sources of anti-cholinesterase enzyme inhibitors. These include Alerianaceae, Amaranthaceae, Amaryllidaceae, Lycopodiaceae Myristicaceae, Polygonaceae, and Rutaceae [35,36]. *R. madagascariensis* belongs to the Strelitziaceae family, and scientific investigations surrounding the potential of this family species are lacking. Hence, this study enabled the shedding of light on the anticholinesterase properties of *R. madagascariensis.* Indeed, it was found that the aqueous extract did not inhibit any of the acetylcholinesterase enzymes (acetylcholinesterase and butyrylcholinesterase). The methanolic extract was the best inhibitor of acetylcholine, while the ethyl acetate extract inhibited butyrylcholinesterase the best.

Today, the new buzz surrounding cosmetics revolves around the inclusion of natural ingredients. Synthetic ingredients are not preferred, since they have a higher tendency to exert allergic effects and other side-effects. The overproduction of the tyrosinase enzyme leads to melanin accumulation which, in turn, is responsible for a wide range of skin conditions including age spots, freckles, hyperpigmentation, sagging and wrinkles [37]. Hence, the inhibition of the tyrosinase enzyme remains important to maintain the beauty and health of the face and the skin in general. All extracts of *R. madagascariensis* could inhibit the tyrosinase enzyme. The ethylacetate extract was the most active, closely followed by the methanolic extract (139.84 ± 0.67 versus 139.08 ± 0.51).

Diabetes mellitus is a disease of carbohydrate, protein and fat metabolism impairment causing hyperglycemia and, therefore, there is a shortage or reduced effectiveness of endogenous insulin. Dysregulated postprandial hyperglycemia in diabetic patients results into diverse health complications such as neuropathy, retinopathy and nephropathy, and some heart diseases. One strategy in reducing postprandial glycemia is to target the alpha-amylase and alpha-glucosidase enzymes in the gastrointestinal tract and retard their carbohydrate breakdown by inhibiting their activities. The ethyl acetate extract was most active (0.85 ± 0.04 mmolACAE/g) against alpha-amylase, followed by the methanolic and aqueous extracts. The methanolic extract was the more effective in inhibiting the alpha-glucosidase enzyme, closely followed by the ethylacetate and aqueous extracts, respectively (1.79 ± 0.01 vs. 1.76 ± 0.02 vs. 1.75 ± 0.01 mmolACAE/g).

In silico methods are employed alongside in vitro data to create a model and to test it. This method comprises databases, homology models, quantitative structure-activity relationships, pharmacophores, and data mining, among other forms of data analysis [38]. In this study, the in silico results demonstrated that metabolites from *R. madagascariensis* are able to bind to the active sites of the enzymes under investigation. Indeed, binding ensued through the generation of various types of chemical bonds, including hydrogen bonds, metallic bonds, ionic and Pi bonds. The binding energies for the identified metabolite in *R. madagascariensis* had noticeable binding energies which are noteworthy and show their affinities for these enzymes and which, at the same time, may complement the inhibition activities of different extracts of this plant species on the different enzymes assayed.

Cancer is a degenerative disease and a leading cause of death. Therapies, especially alternative ones, such as those derived from nature, are being explored urgently in an attempt to use their medicinal properties. Plant-derived bioactive polysaccharides are increasingly being recognized for their antioxidant and anticancer potential, added to the exertion of lower side effects compared to conventional pharmaceuticals [39]. Liver cancer corresponds to 9% of all cancer deaths worldwide [40]. In 2018, gastrointestinal cancer accounted for 26.3% cancer cases, and 35.4% of mortality due to cancer worldwide [41].

Hence, models of liver and gastrointestinal cancer were chosen for this study to investigate the cytotoxicity potential of *R. madagascariensis* extracts on NIH 3T3, HepG2 and HT-29 cell lines. The presence of bioactive polysaccharides in the extracts and the IC_50_ values obtained in the different cytotoxic assays demonstrate the potential of *R. madagascariensis* against cancer cell lines.

## 5. Conclusions

Altogether, 41 metabolites were characterized from the leaf extract of *R. madagascariensis*. Most of the metabolites characterized are well-known for their pharmacological properties. Based on the DPPH, FRAP, CUPRAC, metal chelating and phosphomolybdenum assays, the aqueous extract of *R. madagascariensis* had the best antioxidant properties. The methanolic extract exhibited the most powerful antioxidant activity in the ABTS assay. The methanolic extract was most active against the acetylcholinesterase enzyme, followed by the ethylacetate extract. The anti-tyrosinase activities of the extracts demonstrated that the methanolic and ethyl acetate extracts had almost same efficiencies in inhibiting the tyrosinase enzyme, with the ethyl acetate extract being the most active. All extracts were active against the alpha-amylase and alpha-glucosidase enzymes, with the ethyl acetate extract being the most active against the alpha-amylase enzyme, while the methanolic extract showed the best alpha-glucosidase inhibition. In silico studies demonstrate that the metabolites identified in *R. madagascariensis* have excellent binding affinities with the assayed enzymes, and this may explain the inhibitory effect of this plant on these enzymes. The methanolic extract has good cytotoxic potential against HepG2 and HT 29 cell lines. Based on these results, it can be concluded that the methanolic extract of *R. madagascariensis* should be further explored as a medicinal extract. Additionally, based on the heat map, whereby flavonoids were found to be in greater proportion in the extracts, it can be concluded that the flavonoid portion of the extracts contributed to the most activity. Despite the traditional uses of *R. madagascariensis*, to date no validated dosage schedule for taking this plant species for therapeutic purposes exists. Hence, studies must be geared towards studying the proper dosage for the intake of such a medicinal plant, especially in patients already taking other medicines and in whom herb-drug interactions may be an event. It is recommended that extracts prepared from this plant be considered for the development of nutraceuticals and/or dietary supplements geared towards health and wellness.

## Figures and Tables

**Table 1:** Phytochemical composition.

Extract	Phenolic Content (mg GAE/g)	Total Flavonoid Content (mg RE/g)	Total Phenolic Acid Content (mg CAE/g)	Total Flavonol Content (mg CE/g)
RM-EA	41.13 ± 0.2 ^c^	25.04 ± 0.29 ^b^	7.65 ± 0.52 ^c^	32.22 ± 0.46 ^a^
RM-MEOH	61.06 ± 0.75 ^b^	32.26 ± 0.48 ^a^	16.44 ± 0.47 ^a^	33.73 ± 0.53 ^a^
RM-Aq	64.47 ± 0.19 ^a^	25.30 ± 0.16 ^b^	14.05 ± 0.49 ^b^	2.87 ± 0.08 ^b^

Values are reported as mean ± S.D of three parallel experiments. GAE: Gallic acid equivalent; RE: Rutin equivalent; CAE: Caffeic acid equivalent; CE: Catechin equivalent. Different letters indicate significant differences between the tested extracts (*p* < 0.05, “a” indicates the highest content).

**Table 2:** Characterization of the compounds found in the analyzed extracts of *R. madagascariensis*.

No.	t*_R_* (min)	Observed [M-H]-	Molecular Formula	Error (ppm)	Fragment Ions	Assigned Identification	EA	MeOH	H_2_O
1	1.8	341.1094	C_12_H_22_O_11_	−1.36	**179.0562**, 161.0453, 131.0341, 119.0347, 89.0246	Disaccharide (two hexosides)		✓	
2	2.0	191.0207	C_6_H_8_O_7_	−4.82	173.0092, 129.0193, **111.0086**	Isocitric acid		✓	✓
3	2.7	191.0202	C_6_H_8_O_7_	−2.36	173.0088, 129.0175, **111.0081**	Citric acid		✓	✓
4	3.2	169.0145	C_7_H_6_O_5_	−1.38	**125.0242**	Gallic acid	✓	✓	✓
5	3.6	315.0727	C_13_H_16_O_9_	−2.54	**153.0187**, 109.0294	Dihydroxybenzoic acid-*O*-hexoside		✓	✓
6	5.4	153.0193	C_7_H_6_O_4_	−1.63	**109.0293**	Dihydroxybenzoic acid	✓	✓	✓
7	6.8	175.0615	C_7_H_12_O_5_	−1.92	157.0505, 131.0713, **115.0399**, 85.0661	2-Isopropylmalic acid	✓		✓
8	8.8	137.0242	C_7_H_6_O_3_	1.13	**93.0347**	Salicylic acid	✓	✓	✓
9	12.1	289.0725	C_15_H_14_O_6_	−3.06	**245.0797**, 205.0482, 203.0709, 109.0288	Epicatechin	✓	✓	✓
10	14.4	771.1996	C_33_H_40_O_21_	−1.21	301.0339, **300.0275**, 178.9983, 151.0013	Quercetin-hexoside-rutinoside		✓	✓
11	15.4	273.0777	C_15_H_14_O_5_	−3.16	255.0653, 229.0882, 205.0868, 187.0749, **137.0237**, 107.0494, 97.0291	(Epi)afzelechin	✓	✓	
12	15.8	563.1419	C_26_H_28_O_14_	−2.11	545.1310, 503.1201, 473.1100, 443.0993, 383.0775, **353.0672**	Apigenin-6-*C*-pentoside-8-*C*-hexoside		✓	✓
13	16.5	163.0401	C_9_H_8_O_3_	−0.57	**119.0499**	Coumaric acid	✓	✓	✓
14	17.2	785.2153	C_34_H_42_O_21_	−1.06	**315.0496**, 300.0267, 151.0020	Isorhamnetin-hexoside-rutinoside		✓	✓
15	17.6	545.1467	C_30_H_26_O_10_	−2.43	419.1139, 409.0929, **273.0768**, 164.0108, 125.0243, 97.0297	(Epi)afzelechin-(Epi)afzelechin	✓	✓	✓
16	18.2	545.1470	C_30_H_26_O_10_	−2.89	419.1140, 409.0932, **273.0770**, 164.0113, 125.0241, 97.0292	(Epi)afzelechin-(Epi)afzelechin	✓	✓	✓
17	18.4	739.2099	C_33_H_40_O_19_	−1.01	285.0386, **284.0327**, 257.0454, 255.0291, 227.0342, 178.9996, 151.0027	Kaempferol-deoxyhexoside-rutinoside		✓	✓
18	18.9	933.2302	C_42_H_46_O_24_	0.57	**771.1996**, 631.1852, 301.0345, 300.0286, 178.9988, 151.0019	Quercetin glycoside		✓	✓
19	19.1	639.1580	C_28_H_32_O_17_	−1.81	**315.0486**, 300.0268	Isorhamnetin dihexoside		✓	✓
20	19.9	609.1478	C_27_H_30_O_16_	−2.75	301.0342, **300.0281**, 178.9985, 151.0031	Rutin	✓	✓	✓
21	20.7	463.0891	C_21_H_20_O_12_	−2.03	**301.0351**, 178.9992, 151.0035	Quercetin-hexoside	✓	✓	✓
22	21.5	463.0886	C_21_H_20_O_12_	−2.39	**301.0343**, 178.9979, 151.0029	Quercetin-hexoside	✓	✓	✓
23	22.0	593.1523	C_27_H_30_O_15_	−1.79	285.0396, **284.324**, 255.0305, 227.0343, 151.0043	Kaemferol-rutinoside	✓	✓	✓
24	22.6	817.2146	C_45_H_38_O_15_	−0.83	545.1451, **543.1301**, 419.1144, 273.0762, 271.0616, 164.0117, 125.0232	Proanthocyanidin trimer	✓	✓	✓
25	22.6	947.2466	C_43_H_48_O_24_	−0.25	**771.1975**, 301.0338, 178.9961	Quercetin-glycoside		✓	✓
26	23.1	917.2357	C_42_H_46_O_23_	0.71	**771.1977**, 301.0328, 300.0290, 178.9973, 151.0397	Quercetin-glycoside		✓	✓
27	23.3	593.1523	C_27_H_30_O_15_	−1.70	**285.0403**, 255.0309	Kaemferol-rutinoside		✓	✓
28	23.4	623.1629	C_28_H_32_O_16_	−1.89	**315.0517**, 300.0270, 151.0034	Isorhamnetin-rutinoside	✓	✓	✓
29	24.0	623.1639	C_28_H_32_O_16_	−3.23	477.1082, **315.0517**, 300.0275, 151.0038	Isorhamnetin-deoxyhexoside-hexoside	✓	✓	✓
30	24.2	817.2148	C_45_H_38_O_15_	−0.98	545.1428, **543.1283**, 409.0903, 273.0766, 271.0617, 164.0106, 125.0239	Proanthocyanidin trimer	✓	✓	✓
31	24.6	477.1049	C_22_H_22_O_12_	−2.36	315.0496, **314.0437**, 300.0270, 151.0038	Isorhamentin-hexoside	✓	✓	✓
32	25.3	477.1052	C_22_H_22_O_12_	−3.11	315.0484, **314.0434**, 300.0254, 151.0032	Isorhamentin-hexoside	✓	✓	✓
33	27.1	613.1208	C_29_H_26_O_15_	−1.50	**301.0376**, 178.9995, 151.0033	Quercetin derivative	✓	✓	✓
34	27.4	447.0941	C_21_H_20_O_11_	−1.76	315.0469, **314.0437**, 301.0337, 300.0260, 151.0013	Isorhamnetin-pentoside	✓	✓	✓
35	28.2	625.1211	C_30_H_26_O_15_	−2.04	463.0881, **301.0361**, 178.9991, 151.0033	Quercetin-caffeoylhexoside	✓	✓	✓
36	28.6	545.1465	C_30_H_26_O_10_	−2.25	419.1143, 409.0924, **273.0765**, 164.0113, 125.0238, 97.0289	(Epi)afzelechin-(Epi)afzelechin	✓	✓	✓
37	33.0	639.1355	C_31_H_28_O_15_	−0.91	463.0712, **301.0370**, 178.9976, 151.0045	Quercetin-feruloylhexoside	✓	✓	✓
38	35.6	301.0353	C_15_H_10_O_7_	−1.72	178.9964, **151.0021**	Quercetin	✓	✓	
39	37.7	653.1525	C_32_H_30_O_15_	−1.83	477.1042, **315.0510**, 300.0272, 299.0195	Isorhamnetin-feruloylhexoside	✓	✓	✓
40	38.9	327.2184	C_18_H_32_O_5_	−2.29	291.1993, 229.1430, 211.1325, **171.1020**	Oxo-dihydroxy-octadecenoic acid	✓	✓	✓
41	40.4	329.2340	C_18_H_34_O_5_	−3.00	311.2177, 229.1441, 211.1333, **171.1021**	Trihydroxy-octadecenoic acid	✓	✓	✓

**Table 3:** Antioxidant potential.

Samples	DPPH (mgTE/g)	ABTS (mgTE/g)	FRAP (mgTE/g)	CUPRAC (mgTE/g)	Metal Chelating (mgEDTAE/g)	Phosphomolybdenum (mmolTE/g)
RM-EA	76.43 ± 1.52 ^b^	438.46 ± 1.69 ^c^	128.10 ± 1.49 ^c^	219.81 ± 3.82	5.67 ± 0.26 ^b^	1.78 ± 0.07 ^c^
RM-MEOH	152.28 ± 2.40 ^a^	482.57 ± 0.89 ^a^	205.92 ± 7.24 ^b^	380.14 ± 1.38	6.73 ± 0.14 ^b^	1.98 ± 0.02 ^b^
RM-Aq	154.08 ± 2.43 ^a^	477.02 ± 1.09 ^b^	249.40 ± 3.01 ^a^	384.57 ± 1.99	29.68 ± 0.74 ^a^	2.38 ± 0.07 ^a^

Values are reported as mean ± S.D of three parallel experiments. TE: Trolox equivalent; EDTA: Ethylenediaminetetraacetic acid; EDTAE: EDTA equivalent; DPPH: 2,2-diphenyl-1-picryl-hydrazyl-hydrate; ABTS: 2,2′-azino-bis(3-ethylbenzothiazoline-6-sulfonic acid; FRAP: Ferric reducing antioxidant power; CUPRAC: cupric ion reducing antioxidant capacity. Different letters indicate significant differences between the tested extracts (*p* < 0.05, “a” indicates the highest activity).

**Table 4:** Enzyme inhibitory properties.

Samples	AChE Inhibition (mgGALAE/g)	BchE Inhibition (mgGALAE/g)	Tyrosinase Inhibition (mgKAE/g)	Alpha-Amylase Inhibition (mmolACAE/g)	Alpha-Glucosidase Inhibition (mmolACAE/g)
RM-EA	4.94 ± 0.07 ^a^	6.48 ± 0.62 ^a^	139.84 ± 0.67 ^a^	0.85 ± 0.04 ^a^	1.76 ± 0.02 ^b^
RM-MEOH	5.02 ± 0.06 ^a^	5.27 ± 0.44 ^b^	139.08 ± 0.51 ^a^	0.68 ± 0.03 ^b^	1.79 ± 0.01 ^a^
RM-Aq	na	na	19.36 ± 2.36 ^b^	0.08 ± 0.01 ^c^	1.75 ± 0.01 ^b^

Values are reported as mean ± S.D of three parallel experiments. AChE: Acetylcholinesterase; BChE: Butyrylcholinesterase; GALAE: Galatamine equivalent; KAE: Kojic acid equivalent; ACAE: Acarbose equivalent; RM: *Ravenala madagascariensis* Sonn.; na: not active. Different letters indicate significant differences between the tested extracts (*p* < 0.05, “a” indicates the highest inhibitory effect).

**Table 5:** Docking results.

Metabolite/Pubchem ID	AChE (PDB:2YDM)	Alpha-Amylase (PDB:1VAH)	Alpha-Glucosidase (PDB:3AXI)	Tyrosinase (PDB:2Y9X)	BChE (PDB:5HF5)
	Binding Energies				
2-Isopropylmalic acid/77	−5.1	−5.3	−5.4	−4.7	−5.7
Citric acid/311	−5.5	−5.3	−5.5	−5	−5.7
Salicylic acid/338	−5.6	−4.9	−5.3	−6.1	−6.7
Gallic acid/370	−5.9	−5.3	−5.8	−5.9	−7.1
Isocitric acid/1198	−5.7	−5.4	−5.5	−5.5	−6.1
Epicatechin/72276	−8.3	−6.8	−8.1	−6	−10
(Epi)afzelechin/282014	−7.8	−6.2	−7.4	−6.5	−9.7
Quercetin/5280343	−8.7	−7	−8.6	−6.1	−10.7
Rutin/5280805	−8.4	−8.2	−3	−7.2	−8.8
Isorhamnetin Rutinoside/133562525	−8.4	−7.6	−0.6	−6.8	−4.7
Co-crystal to 2ydm (control)	−6.3				
Co-crystal to 1Vah (control)		−4.9			
Co-crystal to 3axi (control)			−5.6		
Co-crystal to 2y9x (control)				−6.1	
Co-crystal to 5hf5 (control)					−4.5

AChE: Acetylcholinesterase; BChE: butyrylcholinesterase.

**Table 6:** Detailed docking results.

Ligand	Receptor	Residue	Interaction	Distance	E (Kcal/mol)
**Alpha-Amylase-Rutin Docking**					
C9 10	OD1	ASP 300 (A)	H-donor	3.53	−0.6
O14 41	OD1	ASP 356 (A)	H-donor	2.53	−1.0
O7 43	O	HIS 305 (A)	H-donor	3.07	−1.7
O9 66	OD1	ASP 197 (A)	H-donor	2.95	−0.9
O9 66	OD2	ASP 197 (A)	H-donor	2.75	−3.9
O8 68	OE2	GLU 233 (A)	H-donor	2.75	−4.4
O10 64	NE2	HIS 299 (A)	H-acceptor	2.86	−3.5
O4 72	OH	TYR 151 (A)	H-acceptor	2.77	−2.1
**Alpha-Glucosidase-Quercetin Docking**					
O2 27	OE2	GLU 411 (A)	H-donor	2.53	−4.8
O3 29	OD2	ASP 352 (A)	H-donor	2.58	−0.9
O5 31	OD2	ASP 307 (A)	H-donor	2.59	−4.9
O4 2	NE2	GLN 279 (A)	H-acceptor	2.91	−1.1
**Butyrylcholinesterase-Quercetin Docking**					
O7 25	O	TYR 72 (A)	H-donor	2.75	−2.2
O2 27	OG	SER 125 (A)	H-donor	2.78	−2.6
O3 29	OE2	GLU 202 (A)	H-donor	2.50	0.5
O5 31	O	HIS 447 (A)	H-donor	2.61	−2.6
O4 2	N	GLY 121 (A)	H-acceptor	2.90	−2.1
**Acetylcholinesterase-Quercetin Docking**					
O7 25	O	ALA 354 (A)	H-donor	2.99	−2.3
O5 31	OE2	GLU 376 (A)	H-donor	2.53	0.2
ZN 1619	OE 1	GLU 384 (A)	metal	1.76	−5.2
ZN 1619	OE 2	GLU 384 (A)	metal	1.75	−5.2
ZN 1619	OE1	GLU 411 (A)	metal	1.77	−5.3
ZN 1619	OE2	GLU 411 (A)	metal	1.79	−5.2
ZN 1619	OE1	GLU 384 (A)	ionic	1.76	−21.5
ZN 1619	OE2	GLU 384 (A)	ionic	1.75	−21.6
ZN 1619	OE1	GLU 411 (A)	ionic	1.77	−21.2
ZN 1619	OE2	GLU 411 (A)	ionic	1.79	−20.6
6-ring	NE2	GLN 281 (A)	pi-H	4.50	−1.2

## Data Availability

Not applicable.

## Supplementary Materials

The following supporting information can be downloaded at: https://www.mdpi.com/article/10.3390/antiox12010184/s1, Figure S1: Chemical structures of the main compounds found in the analyzed extracts; Table S1: Chemical structures of compounds used for docking studies.
