# Supplementary material for: Above the Invasive and Ornamental Attributes of the Traveler’s Palm: An In Vitro and In Silico Insight into the Anti-Oxidant, Anti-Enzymatic, Cytotoxic and Phytochemical Characterization of Ravenala madagascariensis
Source: Antioxidants (Basel). 2023 Jan 12;12(1):184. doi: 10.3390/antiox12010184 (PMC9854482; doi:10.3390/antiox12010184)
Supplement: Supplementary file 1 [file antioxidants-12-00184-s001.zip › antioxidants-2139320-supplementary.pdf]

Supplementary Information

Above the Invasive and Ornamental Attributes of the Traveler's Palm: An In Vitro and In Silico Insight into the Anti-Oxidant, Anti-Enzymatic, Cytotoxic and Phytochemical Characterization of *Ravenala madagascariensis*

Shanoo Suroowan [1], Eulogio Jose Llorent-Martínez [2], Gokhan Zengin [3], Stefano Dall'Acqua [4,*], Stefania Sut [4], Kalaivani Buskaran [5], Sharida Fakurazi [5,6], Bao Le Van [7,8,*], Mohnad Abdalla [9], Ashraf N. Abdalla [10], Asaad Khalid [11,12] and Mohamad Fawzi Mahomoodally [1,13,14]

[1] Department of Health Sciences, Faculty of Medicine and Health Sciences, University of Mauritius, Réduit 80837, Mauritius; shanoo.suroowan1@umail.uom.ac.mu (S.S.); f.mahomoodally@uom.ac.mu (M.F.M.)
[2] Department of Physical and Analytical Chemistry, University of Jaén, Campus Las Lagunillas S/N, E-23071 Jaén, Spain; ellorent@ujaen.es
[3] Department of Biology, Science Faculty, Selcuk University, Konya 42130, Turkey; gokhanzengin@selcuk.edu.tr
[4] Department of Pharmaceutical and Pharmacological Sciences, University of Padova, Via Marzolo 5, 35131 Padova, Italy; stefania.sut@unipd.it
[5] Laboratory of Natural Medicine and Product Research, Institute of Bioscience, Universiti Putra Malaysia, Serdang 43400, Selangor Darul Ehsan, Malaysia; vaneey_88@yahoo.com (K.B.); sharida@upm.edu.my (S.F.)
[6] Department of Human Anatomy, Faculty of Medicine and Health Sciences, Universiti Putra Malaysia, Serdang 43400, Selangor Darul Ehsan, Malaysia
[7] Institute of Research and Development, Duy Tan University, Da Nang 550000, Vietnam
[8] Faculty of Natural Sciences, Duy Tan University, Da Nang 550000, Vietnam
[9] Pediatric Research Institute, Children's Hospital Affiliated to Shandong University, Jinan 250022, China; mohnadabdalla200@gmail.com
[10] Department of Pharmacology and Toxicology, College of Pharmacy, Umm Al-Qura University, Makkah 21955, Saudi Arabia; anabdrabo@uqu.edu.sa
[11] Substance Abuse and Toxicology Research Center, Jazan University, P.O. Box 114, Jazan 45142, Saudi Arabia; akahmed@jazanu.edu.sa
[12] Medicinal and Aromatic Plants and Traditional Medicine Research Institute, National Center for Research, Khartoum P.O. Box 2404, Sudan
[13] Center for Transdisciplinary Research, Department of Pharmacology, Saveetha Dental College, Saveetha Institute of Medical and Technical Science, Chennai 600077, India
[14] Centre of Excellence for Pharmaceutical Sciences, North-West University, Private Bag X6001, Potchefstroom 2520, South Africa
* Correspondence: stefano.dallacqua@unipd.it (S.D.); vnble@duytan.edu.vn (B.L.V.)

Analysis of phenolic compounds by HPLC-ESI-Q-TOF-MS-MS

For the analysis of compounds, 5 mg of DE were dissolved in 1 mL MeOH or 1 mL 10% MeOH (aqueous extract), filtered through 0.45 µm filters, and 4 µL was injected in the HPLC system. Analyses were performed in an Agilent 1200 (Agilent Technologies, Santa Clara, CA, USA) equipped with an Agilent 6530B quadrupole-time-of-flight mass spectrometer (Q-TOF MS). The column used was a Luna Omega Polar C18 of 150 x 3.0 mm and 5 µm particle size with a Polar C18 Security Guard cartridge (4 x 3.0 mm), both purchased from Phenomenex (Phenomenex, Torrance, CA, USA). The separation was performed at ambient temperature with a gradient elution program at a flow rate of 0.4 mL min^{-1}. The mobile phases consisted of water + formic acid 0.1 % v/v (eluent A) and acetonitrile (eluent B). The gradient elution was: 10-25% B in 0-25 min, 25% B in 25-30 min, 25-50% B in 30-40 min, 50-100% B in 40-42 min, 100% in 42-47 min. Then, eluent B was returned to 10% with a 7 min stabilization time.

To obtain the MS and MS/MS spectra, the mass spectrometer was operated in the negative ion mode using an orthogonal ESI source (Agilent Dual ESI, Santa Clara, CA, USA). The parameters used were: capillary voltage, 3500 V; nebulizer pressure of 45 psi; drying gas flow rate, 10 L/min; gas temperature, 325 °C; skimmer voltage, 60 V; fragmentor voltage, 140 V. Continuous internal calibration was performed during analyses with the use of signals at *m/z* 112.9855 and 1033.9881. The MS and Auto MS/MS modes (using collision energies of 10, 20 and 40 V) were set to acquire m/z values ranging between 50-1200, at a scan rate of 2 and 3 spectra per second, respectively. Agilent Mass Hunter Qualitative analysis software version B.06.00 was used for post-acquisition data processing.

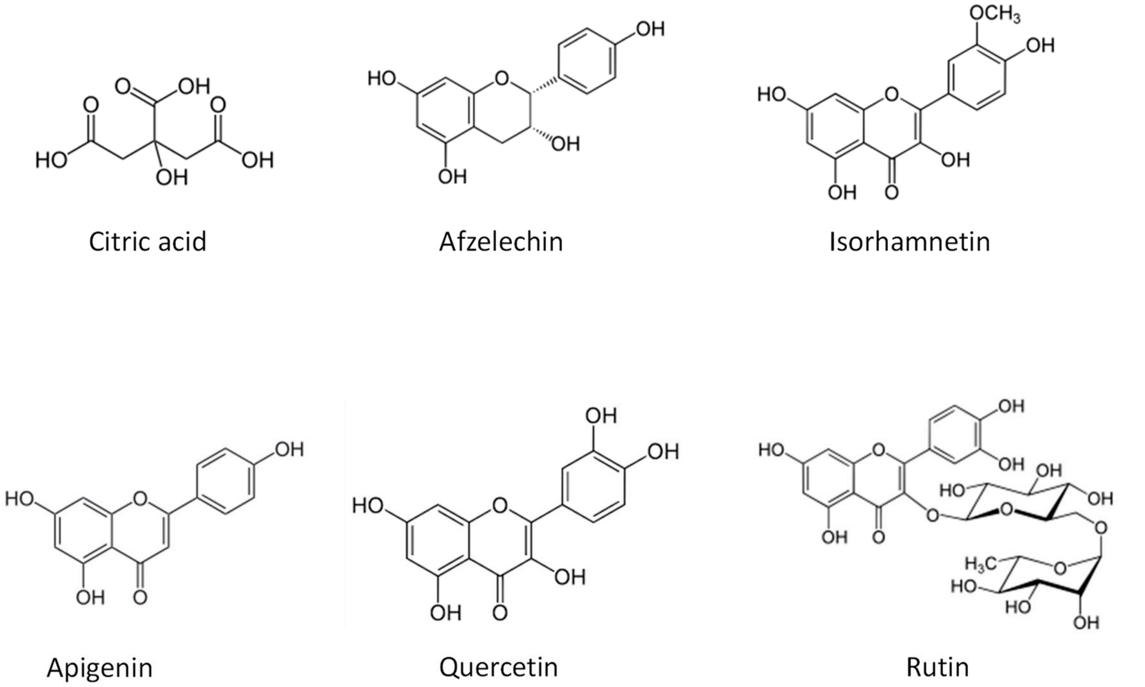

Figure S1: Chemical structures of the main compounds found in the analyzed extracts

Table S1: Chemical structures of compounds used for docking studies

Compound	IUPAC Name	Pubchem database link	PubChem CID
(Epi)afzelechin	2-(4-Hydroxyphenyl)-3,4-dihydro-2H-1-benzopyran-3,5,7-triol	2-(4-Hydroxyphenyl)-3,4-dihydro-2H-1-benzopyran-3,5,7-triol | C15H14O5 - PubChem (nih.gov)	282014
2-Isopropylmalic acid	2-hydroxy-2-propan-2-ylbutanedioic acid	2-Isopropylmalic acid | C7H12O5 - PubChem (nih.gov)	77
Citric acid	2-hydroxypropane-1,2,3-tricarboxylic acid	Citric Acid | C6H8O7 - PubChem (nih.gov)	311
Coutaric acid	(2*R*,3*R*)-2-hydroxy-3-[(*E*)-3-(4-hydroxyphenyl)prop-2-enoyl]oxybutanedioic acid	Coutaric acid | C13H12O8 - PubChem (nih.gov)	57517924
Epicatechin	(2*R*,3*R*)-2-(3,4-dihydroxyphenyl)-3,4-dihydro-2*H*-chromene-3,5,7-triol	(-)-Epicatechin | C15H14O6 - PubChem (nih.gov)	72276

Ferulic acid	(*E*)-3-(4-hydroxy-3-methoxyphenyl)prop-2-enoic acid	Ferulic acid | C10H10O4 - PubChem (nih.gov)	445858
Gallic acid	3,4,5-trihydroxybenzoic acid	Gallic Acid | C7H6O5 - PubChem (nih.gov)	370
Isocitric acid	1-hydroxypropane-1,2,3-tricarboxylic acid	Isocitric acid | C6H8O7 - PubChem (nih.gov)	1198
Isorhamnetin Rutinoside	5,7-dihydroxy-2-(4-hydroxy-3-methoxyphenyl)-3-[(2*S*,3*R*,4*S*,5*S*)-3,4,5-trihydroxy-6-[[(2*R*,3*R*,4*R*,5*R*,6*S*)-3,4,5-trihydroxy-6-methyloxan-2-yl]oxymethyl]oxan-2-yl]oxychromen-4-one	Isorhamnetin rutinoside | C28H32O16 - PubChem (nih.gov)	133562525
Kiwiionoside	2-[3,4-dihydroxy-4-[(*E*)-3-hydroxybut-1-enyl]-3,5,5-trimethylcyclohexyl]oxy-6-(hydroxymethyl)oxane-3,4,5-triol	Kiwiionoside | C19H34O9 - PubChem (nih.gov)	131752431
N-Feruloyltyramine	(*E*)-3-(4-hydroxy-3-methoxyphenyl)-*N*-[2-(4-hydroxyphenyl)ethyl]prop-2-enamide	Moupinamide | C18H19NO4 - PubChem (nih.gov)	5280537
Phlorizin	1-[2,4-dihydroxy-6-[(2*S*,3*R*,4*S*,5*S*,6*R*)-3,4,5-trihydroxy-6-(hydroxymethyl)oxan-2-yl]oxyphenyl]-3-(4-hydroxyphenyl)propan-1-one	Phlorizin | C21H24O10 - PubChem (nih.gov)	6072
Quercetin	2-(3,4-dihydroxyphenyl)-3,5,7-trihydroxychromen-4-one	Quercetin | C15H10O7 - PubChem (nih.gov)	5280343
Roseoside	(4*S*)-4-hydroxy-3,5,5-trimethyl-4-[(*E*,3*R*)-3-[(2*R*,3*R*,4*S*,5*S*,6*R*)-3,4,5-trihydroxy-6-(hydroxymethyl)oxan-2-yl]oxybut-1-enyl]cyclohex-2-en-1-one	Roseoside | C19H30O8 - PubChem (nih.gov)	9930064
Rutin	2-(3,4-dihydroxyphenyl)-5,7-dihydroxy-3-[(2*S*,3*R*,4*S*,5*S*,6*R*)-3,4,5-trihydroxy-6-[[(2*R*,3*R*,4*R*,5*R*,6*S*)-3,4,5-trihydroxy-6-methyloxan-2-yl]oxymethyl]oxan-2-yl]oxychromen-4-one	Rutin | C27H30O16 - PubChem (nih.gov)	5280805
Salicylic acid	2-hydroxybenzoic acid	Salicylic Acid | HOC6H4COOH - PubChem (nih.gov)	338
